# 2,9-Dimethyl-4,7-diphenyl-1,10-phenanthrolin-1-ium tetra­chloridoaurate(III)

**DOI:** 10.1107/S1600536809011994

**Published:** 2009-04-02

**Authors:** Sema Öztürk Yıldırım, Mehmet Akkurt, Nasser Safari, Anita Abedi, Vahid Amani, Vickie McKee

**Affiliations:** a Department of Physics, Faculty of Arts and Sciences, Erciyes University, 38039 Kayseri, Turkey; b Department of Chemistry, Shahid Beheshti University, GC, Evin, Tehran 1983963113, Iran; c Department of Chemistry, North Tehran Branch, Islamic Azad University, Tehran, Iran; d Chemistry Department, Loughborough University, Loughborough, Leicestershire LE11 3TU, England

## Abstract

Both the cation and anion of the title compound, (C_26_H_21_N_2_)[AuCl_4_], are disposed about a plane of mirror symmetry. The 2,9-dimethyl-4,7-diphenyl-1,10-phenanthrolinium ring is oriented at a dihedral angle of 44.2 (1)° with respect to the planar phenyl ring systems. The Au^III^ atom has a square-planar environment defined by four Cl atoms. The crystal structure is stabilized by C—H⋯π and Au⋯π ring–metal (3.551 Å) inter­actions. In the crystal structure, the mol­ecules stack along the *c* axis *via* N—H⋯N hydrogen-bond inter­actions.

## Related literature

For general background to proton-transfer systems and their structures, see: Abedi *et al.* (2008 ▶); Amani *et al.* (2008 ▶); Calleja *et al.* (2001 ▶); Hasan *et al.* (1999 ▶); Hojjat Kashani *et al.* (2008 ▶); Johnson & Steed (1998 ▶); Karaca *et al.* (2009 ▶); Yap *et al.* (1995 ▶); Zhang *et al.* (2006 ▶).
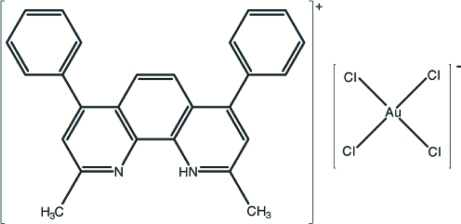


## Experimental

### 

#### Crystal data

(C_26_H_21_N_2_)[AuCl_4_]
*M* *_r_* = 700.22
Orthorhombic, 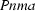

*a* = 13.5195 (10) Å
*b* = 22.9565 (17) Å
*c* = 7.5556 (6) Å
*V* = 2345.0 (3) Å^3^
*Z* = 4
Mo *K*α radiation
μ = 6.75 mm^−1^
*T* = 150 K
0.20 × 0.06 × 0.04 mm

#### Data collection

Bruker APEXII CCD diffractometer
Absorption correction: multi-scan (*SADABS*; Sheldrick, 2003 ▶) *T*_min_ = 0.345, *T*_max_ = 0.774
24337 measured reflections
3265 independent reflections
2835 reflections with *I* > 2σ(*I*)
*R*_int_ = 0.046

#### Refinement

*R*[*F* ^2^ > 2σ(*F* ^2^)] = 0.023
*wR*(*F* ^2^) = 0.054
*S* = 1.03
3265 reflections
159 parameters
H atoms treated by a mixture of independent and constrained refinement
Δρ_max_ = 0.85 e Å^−3^
Δρ_min_ = −0.60 e Å^−3^

### 

Data collection: *APEX2* (Bruker, 2005 ▶); cell refinement: *SAINT* (Bruker, 2005 ▶); data reduction: *SAINT*; program(s) used to solve structure: *SIR97* (Altomare *et al.*, 1999 ▶); program(s) used to refine structure: *SHELXL97* (Sheldrick, 2008 ▶); molecular graphics: *ORTEP-3 for Windows* (Farrugia, 1997 ▶); software used to prepare material for publication: *WinGX* (Farrugia, 1999 ▶).

## Supplementary Material

Crystal structure: contains datablocks global, I. DOI: 10.1107/S1600536809011994/hg2496sup1.cif

Structure factors: contains datablocks I. DOI: 10.1107/S1600536809011994/hg2496Isup2.hkl

Additional supplementary materials:  crystallographic information; 3D view; checkCIF report

## Figures and Tables

**Table 1:** Hydrogen-bond geometry (Å, °)

*D*—H⋯*A*	*D*—H	H⋯*A*	*D*⋯*A*	*D*—H⋯*A*
N1—H*N*1⋯N1^i^	0.76 (5)	2.28 (5)	2.646 (3)	111 (4)
C1—H1*B*⋯*Cg*3^ii^	0.96	2.76	3.574 (3)	143

Symmetry codes: (i) 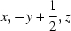; (ii) 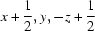. *Cg*3 is the centroid of the C8–C13 ring.

**Fig. 1.**
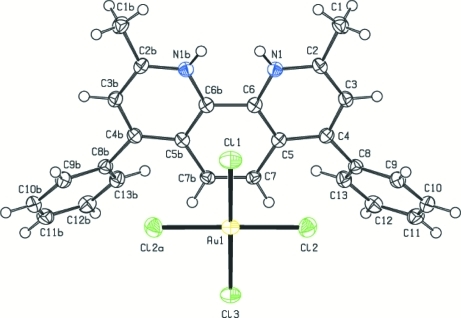
ORTEP drawing of the title compound with the atom numbering scheme. Displacement ellipsoids for non-H atoms are drawn at the 50% probability level [Symmetry codes: (a and b) x, 1/2 - y, z].

**Fig. 2.**
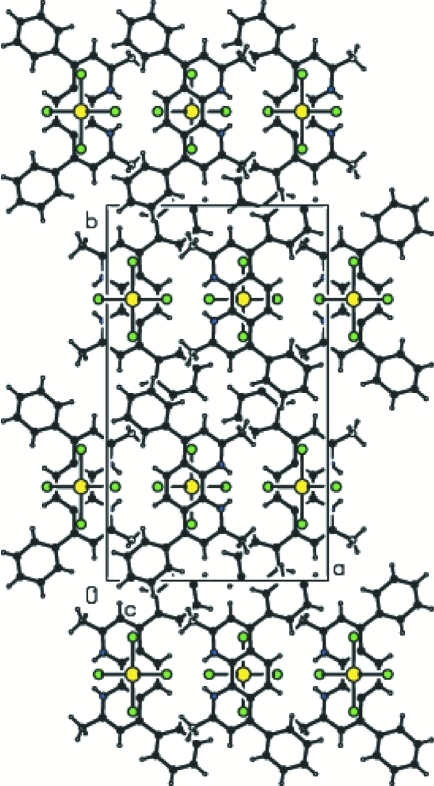
The packing of the title compound viewed down c axis.

## supplementary crystallographic information

### Comment

In recent years, there has been considerable interest in proton transfer systems and their structures (Amani *et al.*, 2008; Abedi *et al.*, 2008; Karaca *et al.*, 2009). Several proton transfer systems using HAuCl_4_ with proton acceptor molecules, such as [EMI][AuCl_4_] and [BMI]_2_[AuCl_4_].2H_2_O (Hasan *et al.*, 1999), [H_2_bipy][AuCl_4_][Cl] (Zhang *et al.*, 2006), [H_7_O_3_][15-crown-5][AuCl_4_] and [H_5_O_2_][benzo-15-crown-5]_2_[AuCl_4_] (Johnson & Steed, 1998), [H_5_O_2_]_2_[12-crown-4]_2_[AuCl_4_]_2, _ [H_3_O][18-crown-6][AuCl_4_] and [H_3_O][4-nitrobenzo-18-crown-6][AuCl_4_] (Calleja *et al.*, 2001), [DPpy.H][AuCl_4_] (Yap *et al.*, 1995) and [H_2_DA18C6][AuCl_4_].2H_2_O (Hojjat Kashani *et al.*, 2008) [where EMI is 1-ethyl-3-methylimidazolium,BMI is 1-butyl-3-methylimidazolium, H_2_bipy is 2, 2'-bipyridinium, DPpy.H is 2,6-diphenylpyridinium and H_2_DA18C6 is 1,10-diazonia-18-crown-6] have been synthesized and characterized by single-crystal X-ray diffraction methods. We report herein the synthesis and crystal structure of the title compound, (I).

Both the cation and anion of the title compound (Fig. 1) are disposed about a plane of mirror symmetry. The 2,9-dimethyl-4,7-diphenyl-1,10-phenanthroline ring is oriented at a dihedral angle of 44.2 (1)° with respect to the planar phenyl ring systems. The Au ion has a square-planar environment defined by four Cl atoms. In AuCl_4_ anion, the Au—Cl bond lengths and Cl—Au—Cl bond angles are normal ranges. In the crystal structure, there exist ring-metal interactions [*Cg*2···Au1(*x*, *y*, *z*) = 3.551 Å and *Cg*2···Au1(*x*, 1/2 - *y*, *z*) = 3.551 Å, where *Cg*2 is a centroid of the central benzene ring (C5–C7/C5b–C7b) of the cation molecule]. For C1—H1B···*Cg*3 interaction, C1···*Cg*3 = 3.574Å, where *Cg*3 is a centroid of the phenyl ring (C8 –C13) (Table 2). View of the packing of (I) down the c-axis is given in Fig. 2.

### Experimental

For the preparation of the title compound, a solution of 2,9-dimethyl-4,7-diphenyl-1,10-phenanthroline (0.15 g, 0.44 mmol) in HCCl_3_ (10 ml) was added to a solution of HAuCl_4_.3H_2_O, (0.17 g, 0.44 mmol) in ethanol (5 ml) and the resulting yellow solution was stirred for 15 min at 313 K. This solution was left to evaporate slowly at room temperature. After one week, yellow prismatic crystals of the title compound were isolated [yield; 0.23 g; 72.1%; m. p. 495 K].

### Refinement

All H atoms were seen in the difference electron density map. The H atom HN1 bound to atom N1 were refined isotropically. Since the anion group of the title molecule has symmetrically two parts, the site occupation factor of the HN1 atom were fixed at the value of 0.5. The other H atoms were positioned geometrically and refined using a riding model, with C—H = 0.93 and 0.96 Å and with *U*_iso_(H) = 1.2 or 1.5*U*_eq_(C). The highest residual peak is located 0.87 Å from atom Au1 and the deepest hole is located 0.57 Å from atom Au1.

### Crystal data

**Table:**

(C_26_H_21_N_2_)[AuCl_4_]	*F*(000) = 1352
*M**_r_* = 700.22	*D*_x_ = 1.983 Mg m^−^^3^
Orthorhombic, *P**n**m**a*	Mo *K*α radiation, λ = 0.71069 Å
Hall symbol: -P 2ac 2n	Cell parameters from 5918 reflections
*a* = 13.5195 (10) Å	θ = 2.8–27.6°
*b* = 22.9565 (17) Å	µ = 6.75 mm^−^^1^
*c* = 7.5556 (6) Å	*T* = 150 K
*V* = 2345.0 (3) Å^3^	Prism, yellow
*Z* = 4	0.20 × 0.06 × 0.04 mm

### Data collection

**Table:**

Bruker APEXII CCD diffractometer	3265 independent reflections
Radiation source: sealed tube	2835 reflections with *I* > 2σ(*I*)
graphite	*R*_int_ = 0.046
φ and ω scans	θ_max_ = 29.2°, θ_min_ = 1.8°
Absorption correction: multi-scan (*SADABS*; Sheldrick, 2003)	*h* = −18→18
*T*_min_ = 0.345, *T*_max_ = 0.774	*k* = −31→31
24337 measured reflections	*l* = −10→10

### Refinement

**Table:**

Refinement on *F*^2^	Primary atom site location: structure-invariant direct methods
Least-squares matrix: full	Secondary atom site location: difference Fourier map
*R*[*F*^2^ > 2σ(*F*^2^)] = 0.023	Hydrogen site location: inferred from neighbouring sites
*wR*(*F*^2^) = 0.054	H atoms treated by a mixture of independent and constrained refinement
*S* = 1.03	*w* = 1/[σ^2^(*F*_o_^2^) + (0.026*P*)^2^ + 1.1102*P*] where *P* = (*F*_o_^2^ + 2*F*_c_^2^)/3
3265 reflections	(Δ/σ)_max_ = 0.001
159 parameters	Δρ_max_ = 0.85 e Å^−^^3^
0 restraints	Δρ_min_ = −0.60 e Å^−^^3^

### Special details

**Table:**

Geometry. Bond distances, angles *etc*. have been calculated using the rounded fractional coordinates. All su's are estimated from the variances of the (full) variance-covariance matrix. The cell e.s.d.'s are taken into account in the estimation of distances, angles and torsion angles
Refinement. Refinement on *F*^2^ for ALL reflections except those flagged by the user for potential systematic errors. Weighted *R*-factors *wR* and all goodnesses of fit *S* are based on *F*^2^, conventional *R*-factors *R* are based on *F*, with *F* set to zero for negative *F*^2^. The observed criterion of *F*^2^ > σ(*F*^2^) is used only for calculating -*R*-factor-obs *etc*. and is not relevant to the choice of reflections for refinement. *R*-factors based on *F*^2^ are statistically about twice as large as those based on *F*, and *R*-factors based on ALL data will be even larger.

### Fractional atomic coordinates and isotropic or equivalent isotropic displacement parameters (Å^2^)

**Table:**

	*x*	*y*	*z*	*U*_iso_*/*U*_eq_	Occ. (<1)
N1	0.51546 (16)	0.19238 (10)	0.0058 (3)	0.0203 (7)	
C1	0.61127 (19)	0.10789 (13)	−0.0819 (4)	0.0271 (8)	
C2	0.52022 (19)	0.13465 (11)	−0.0049 (3)	0.0202 (8)	
C3	0.4389 (2)	0.10119 (12)	0.0488 (4)	0.0219 (8)	
C4	0.3533 (2)	0.12606 (12)	0.1132 (3)	0.0200 (7)	
C5	0.35028 (19)	0.18757 (11)	0.1303 (3)	0.0181 (7)	
C6	0.43300 (19)	0.21879 (11)	0.0701 (3)	0.0190 (7)	
C7	0.27048 (18)	0.22031 (10)	0.2068 (3)	0.0182 (7)	
C8	0.26628 (19)	0.08820 (11)	0.1485 (3)	0.0199 (7)	
C9	0.2791 (2)	0.03400 (11)	0.2301 (4)	0.0237 (8)	
C10	0.2004 (2)	−0.00427 (12)	0.2445 (4)	0.0272 (8)	
C11	0.1082 (2)	0.01067 (13)	0.1803 (4)	0.0279 (8)	
C12	0.0937 (2)	0.06484 (13)	0.1013 (4)	0.0263 (8)	
C13	0.1724 (2)	0.10337 (12)	0.0840 (3)	0.0230 (8)	
Au1	0.38322 (1)	0.25000	0.60223 (2)	0.0219 (1)	
Cl1	0.53754 (7)	0.25000	0.48086 (15)	0.0343 (3)	
Cl2	0.38270 (5)	0.15042 (4)	0.60263 (10)	0.0336 (2)	
Cl3	0.22961 (7)	0.25000	0.72842 (14)	0.0298 (3)	
HN1	0.556 (4)	0.212 (2)	−0.029 (7)	0.009 (14)*	0.500
H1A	0.59680	0.09310	−0.19790	0.0410*	
H1B	0.63310	0.07660	−0.00740	0.0410*	
H1C	0.66240	0.13680	−0.08990	0.0410*	
H3	0.44280	0.06080	0.04060	0.0260*	
H7	0.21760	0.20050	0.25760	0.0220*	
H9	0.34070	0.02370	0.27490	0.0280*	
H10	0.20980	−0.04040	0.29790	0.0330*	
H11	0.05590	−0.01540	0.18980	0.0340*	
H12	0.03130	0.07520	0.06010	0.0310*	
H13	0.16280	0.13930	0.02960	0.0280*	

### Atomic displacement parameters (Å^2^)

**Table:**

	*U*^11^	*U*^22^	*U*^33^	*U*^12^	*U*^13^	*U*^23^
N1	0.0164 (11)	0.0215 (11)	0.0230 (12)	−0.0001 (9)	0.0006 (9)	0.0006 (9)
C1	0.0232 (14)	0.0249 (14)	0.0333 (16)	0.0047 (11)	0.0024 (12)	−0.0025 (12)
C2	0.0215 (13)	0.0200 (13)	0.0191 (13)	0.0039 (10)	−0.0020 (10)	−0.0022 (10)
C3	0.0242 (14)	0.0194 (13)	0.0220 (13)	0.0028 (10)	−0.0005 (10)	−0.0008 (10)
C4	0.0204 (12)	0.0205 (12)	0.0192 (12)	−0.0002 (10)	−0.0012 (10)	0.0019 (10)
C5	0.0176 (11)	0.0199 (12)	0.0167 (12)	0.0000 (10)	−0.0030 (9)	0.0014 (9)
C6	0.0186 (12)	0.0205 (13)	0.0180 (12)	0.0000 (10)	−0.0032 (9)	0.0014 (10)
C7	0.0174 (11)	0.0204 (12)	0.0168 (12)	−0.0035 (9)	0.0008 (9)	−0.0002 (10)
C8	0.0207 (12)	0.0198 (12)	0.0193 (12)	−0.0009 (10)	0.0004 (10)	−0.0022 (10)
C9	0.0226 (12)	0.0229 (13)	0.0256 (14)	0.0033 (10)	0.0005 (11)	0.0017 (11)
C10	0.0325 (15)	0.0208 (13)	0.0284 (15)	0.0018 (11)	0.0057 (12)	0.0040 (11)
C11	0.0292 (14)	0.0266 (14)	0.0280 (15)	−0.0071 (12)	0.0058 (12)	−0.0028 (12)
C12	0.0218 (13)	0.0292 (15)	0.0278 (14)	0.0010 (11)	−0.0015 (11)	−0.0031 (12)
C13	0.0246 (14)	0.0210 (13)	0.0233 (13)	0.0025 (10)	−0.0018 (11)	−0.0004 (10)
Au1	0.0177 (1)	0.0292 (1)	0.0187 (1)	0.0000	−0.0022 (1)	0.0000
Cl1	0.0186 (4)	0.0474 (6)	0.0370 (6)	0.0000	0.0020 (4)	0.0000
Cl2	0.0326 (4)	0.0297 (4)	0.0386 (4)	0.0049 (3)	0.0006 (3)	0.0074 (3)
Cl3	0.0212 (4)	0.0383 (6)	0.0298 (5)	0.0000	0.0043 (4)	0.0000

### Geometric parameters (Å, °)

**Table:**

Au1—Cl2^i^	2.2860 (9)	C8—C13	1.404 (4)
Au1—Cl3	2.2852 (10)	C8—C9	1.399 (4)
Au1—Cl1	2.2790 (10)	C9—C10	1.384 (4)
Au1—Cl2	2.2860 (9)	C10—C11	1.381 (4)
N1—C2	1.329 (3)	C11—C12	1.393 (4)
N1—C6	1.359 (3)	C12—C13	1.390 (4)
N1—HN1	0.76 (5)	C1—H1B	0.9600
C1—C2	1.494 (4)	C1—H1C	0.9600
C2—C3	1.401 (4)	C1—H1A	0.9600
C3—C4	1.379 (4)	C3—H3	0.9300
C4—C5	1.419 (4)	C7—H7	0.9300
C4—C8	1.487 (4)	C9—H9	0.9300
C5—C6	1.404 (4)	C10—H10	0.9300
C5—C7	1.436 (3)	C11—H11	0.9300
C6—C6^i^	1.433 (4)	C12—H12	0.9300
C7—C7^i^	1.363 (3)	C13—H13	0.9300
			
Au1···C7^i^	3.423 (2)	C13···C7	3.135 (4)
Au1···C7	3.423 (2)	C3···H10^xii^	3.1000
Cl1···Cl3^ii^	3.4011 (15)	C3···H9	2.8000
Cl1···C7^iii^	3.520 (3)	C5···H13	2.8700
Cl1···Cl2	3.2333 (11)	C7···H13	2.7100
Cl1···C6	3.485 (3)	C8···H10^xii^	2.8900
Cl1···Cl3^iv^	3.4011 (15)	C8···H7	2.7900
Cl1···C7^v^	3.520 (3)	C9···H3	2.7100
Cl1···Cl2^i^	3.2333 (11)	C9···H1B^ix^	3.0400
Cl1···C6^i^	3.485 (3)	C10···H1B^ix^	2.8700
Cl2···Cl1	3.2333 (11)	C11···H1B^ix^	2.9200
Cl2···C3^vi^	3.636 (3)	C13···H10^xii^	3.0500
Cl2···Cl3	3.2269 (11)	C13···H7	2.6600
Cl2···C2^vi^	3.519 (2)	HN1···Cl3^iii^	2.92 (5)
Cl3···Cl1^vii^	3.4011 (15)	HN1···H1C	2.2900
Cl3···Cl2	3.2269 (11)	HN1···Cl3^v^	2.92 (5)
Cl3···Cl1^viii^	3.4011 (15)	HN1···N1^i^	2.28 (5)
Cl3···Cl2^i^	3.2269 (11)	H1B···C11^iii^	2.9200
Cl1···H13^v^	3.0500	H1B···C10^iii^	2.8700
Cl1···H13^iii^	3.0500	H1B···C9^iii^	3.0400
Cl2···H12^iii^	2.9200	H1C···HN1	2.2900
Cl2···H1C^ix^	3.0000	H1C···Cl3^v^	2.9500
Cl3···HN1^x^	2.92 (5)	H1C···Cl2^iii^	3.0000
Cl3···HN1^ix^	2.92 (5)	H1C···Cl3^iii^	2.9500
Cl3···H1C^x^	2.9500	H3···H9	2.4000
Cl3···H1C^ix^	2.9500	H3···C9	2.7100
N1···N1^i^	2.646 (3)	H7···C13	2.6600
N1···HN1^i^	2.28 (5)	H7···C8	2.7900
C2···C12^iii^	3.585 (4)	H7···H13	2.3400
C2···Cl2^xi^	3.519 (2)	H9···H3	2.4000
C3···C12^iii^	3.474 (4)	H9···C3	2.8000
C3···Cl2^xi^	3.636 (3)	H10···C13^xiii^	3.0500
C6···Cl1	3.485 (3)	H10···C3^xiii^	3.1000
C6···Cl1	3.485 (3)	H10···C8^xiii^	2.8900
C7···C13	3.135 (4)	H12···Cl2^ix^	2.9200
C7···Au1	3.423 (2)	H13···C7	2.7100
C7···Au1	3.423 (2)	H13···H7	2.3400
C7···Cl1^ix^	3.520 (3)	H13···Cl1^x^	3.0500
C7···Cl1^x^	3.520 (3)	H13···Cl1^ix^	3.0500
C12···C3^ix^	3.474 (4)	H13···C5	2.8700
C12···C2^ix^	3.585 (4)		
			
Cl1—Au1—Cl3	179.07 (4)	C8—C9—C10	120.3 (3)
Cl1—Au1—Cl2^i^	90.19 (2)	C9—C10—C11	120.6 (3)
Cl2—Au1—Cl3	89.81 (2)	C10—C11—C12	120.0 (3)
Cl2—Au1—Cl2^i^	179.62 (3)	C11—C12—C13	120.0 (3)
Cl2^i^—Au1—Cl3	89.81 (2)	C8—C13—C12	120.1 (2)
Cl1—Au1—Cl2	90.19 (2)	H1A—C1—H1C	109.00
C2—N1—C6	120.4 (2)	C2—C1—H1A	109.00
C6—N1—HN1	117 (4)	C2—C1—H1B	109.00
C2—N1—HN1	123 (4)	C2—C1—H1C	109.00
C1—C2—C3	122.3 (2)	H1A—C1—H1B	109.00
N1—C2—C3	119.4 (2)	H1B—C1—H1C	109.00
N1—C2—C1	118.3 (2)	C2—C3—H3	119.00
C2—C3—C4	122.3 (3)	C4—C3—H3	119.00
C3—C4—C8	119.0 (2)	C7^i^—C7—H7	119.00
C5—C4—C8	122.9 (2)	C5—C7—H7	119.00
C3—C4—C5	117.9 (2)	C8—C9—H9	120.00
C6—C5—C7	117.5 (2)	C10—C9—H9	120.00
C4—C5—C7	125.4 (2)	C9—C10—H10	120.00
C4—C5—C6	117.1 (2)	C11—C10—H10	120.00
C5—C6—C6^i^	120.7 (2)	C12—C11—H11	120.00
N1—C6—C6^i^	116.5 (2)	C10—C11—H11	120.00
N1—C6—C5	122.8 (2)	C11—C12—H12	120.00
C5—C7—C7^i^	121.6 (2)	C13—C12—H12	120.00
C4—C8—C13	120.6 (2)	C12—C13—H13	120.00
C9—C8—C13	119.1 (2)	C8—C13—H13	120.00
C4—C8—C9	120.1 (2)		
			
C6—N1—C2—C1	178.1 (2)	C7—C5—C6—N1	175.7 (2)
C6—N1—C2—C3	0.8 (4)	C7—C5—C6—C6^i^	−5.8 (3)
C2—N1—C6—C5	1.0 (4)	C4—C5—C7—C7^i^	−175.3 (2)
C2—N1—C6—C6^i^	−177.6 (2)	C6—C5—C7—C7^i^	5.9 (3)
N1—C2—C3—C4	−0.1 (4)	N1—C6—C6^i^—N1^i^	0.0 (3)
C1—C2—C3—C4	−177.3 (3)	N1—C6—C6^i^—C5^i^	178.6 (2)
C2—C3—C4—C5	−2.2 (4)	C5—C6—C6^i^—N1^i^	−178.6 (2)
C2—C3—C4—C8	173.2 (2)	C5—C6—C6^i^—C5^i^	0.0 (4)
C3—C4—C5—C6	3.7 (3)	C5—C7—C7^i^—C5^i^	0.0 (4)
C3—C4—C5—C7	−175.1 (2)	C4—C8—C9—C10	−172.4 (2)
C8—C4—C5—C6	−171.5 (2)	C13—C8—C9—C10	0.9 (4)
C8—C4—C5—C7	9.6 (4)	C4—C8—C13—C12	173.2 (2)
C3—C4—C8—C9	42.3 (3)	C9—C8—C13—C12	−0.1 (4)
C3—C4—C8—C13	−130.8 (3)	C8—C9—C10—C11	−0.6 (4)
C5—C4—C8—C9	−142.5 (3)	C9—C10—C11—C12	−0.4 (5)
C5—C4—C8—C13	44.4 (3)	C10—C11—C12—C13	1.3 (4)
C4—C5—C6—N1	−3.2 (3)	C11—C12—C13—C8	−1.0 (4)
C4—C5—C6—C6^i^	175.3 (2)		

Symmetry codes: (i) *x*, −*y*+1/2, *z*; (ii) *x*+1/2, *y*, −*z*+3/2; (iii) *x*+1/2, *y*, −*z*+1/2; (iv) *x*+1/2, −*y*+1/2, −*z*+3/2; (v) *x*+1/2, −*y*+1/2, −*z*+1/2; (vi) *x*, *y*, *z*+1; (vii) *x*−1/2, *y*, −*z*+3/2; (viii) *x*−1/2, −*y*+1/2, −*z*+3/2; (ix) *x*−1/2, *y*, −*z*+1/2; (x) *x*−1/2, −*y*+1/2, −*z*+1/2; (xi) *x*, *y*, *z*−1; (xii) −*x*+1/2, −*y*, *z*−1/2; (xiii) −*x*+1/2, −*y*, *z*+1/2.

### Hydrogen-bond geometry (Å, °)

**Table:**

*D*—H···*A*	*D*—H	H···*A*	*D*···*A*	*D*—H···*A*
N1—HN1···N1^i^	0.76 (5)	2.28 (5)	2.646 (3)	111 (4)
C1—H1B···Cg3^iii^	0.96	2.76	3.574 (3)	143

Symmetry codes: (i) *x*, −*y*+1/2, *z*; (iii) *x*+1/2, *y*, −*z*+1/2.
